# Corrosion Inhibition of High Speed Steel by Biopolymer HPMC Derivatives

**DOI:** 10.3390/ma9080612

**Published:** 2016-07-23

**Authors:** Shih-Chen Shi, Chieh-Chang Su

**Affiliations:** Department of Mechanical Engineering, National Cheng Kung University (NCKU), No. 1 University Road, Tainan 70101, Taiwan

**Keywords:** corrosion, HSS, biopolymer, HPMC, green inhibitor

## Abstract

The corrosion inhibition characteristics of the derivatives of biopolymer hydroxypropyl methylcellulose (HPMC), hydroxypropyl methylcellulose phthalate (HPMCP), and hydroxypropyl methylcellulose acetate succinate (HPMCAS) film are investigated. Based on electrochemical impedance spectroscopic measurements and potentiodynamic polarization, the corrosion inhibition performance of high speed steel coated with HPMC derivatives is evaluated. The Nyquist plot and Tafel polarization demonstrate promising anti-corrosion performance of HPMC and HPMCP. With increasing film thickness, both materials reveal improvement in corrosion inhibition. Moreover, because of a hydrophobic surface and lower moisture content, HPMCP shows better anti-corrosion performance than HPMCAS. The study is of certain importance for designing green corrosion inhibitors of high speed steel surfaces by the use of biopolymer derivatives.

## 1. Introduction

Currently, there are three primary methods of corrosion control: reducing metal oxidation [1], decreasing corrosion of corrosive liquids [2], and isolating metal from the corrosive environment via dry films [3,4,5]. In a liquid environment, the common approach is to add corrosion inhibitors to form a physical adsorption layer on the surface, thereby blocking the penetration of active substances and reducing corrosion [6,7,8]. However, most inhibitors are harmful and can contaminate the global environment. Thus, several researchers have investigated the effects of polymeric corrosion inhibitors, such as polyamide compounds [9], polyacrylic acid [10], polymeric materials [11], and cellulosic polymers [12], which show considerable promised anticorrosion behavior.

According to the requirements of the Paris climate agreements and sustainable development, extensive studies on green materials for anti-corrosive films have recently been conducted [13,14,15,16]. The objective of the present study is to evaluate the anti-corrosion performance of biopolymer hydroxypropyl methylcellulose (HPMC) derivatives in a saline solution. HPMC was extracted from high purity wood pulp from natural forests. Owing to its high film forming ability [17] and flexibility [18], it has been widely employed in the medical field [19,20,21] and the food industry [22]. Meanwhile, HPMC has a good capability for distributing and preventing grease and gas penetration [23], and is therefore used in sustainable manufacturing [24]. Moreover, owing to its biocompatibility [25] and decomposability [26,27], it is also used as a corrosion inhibitor [28,29,30,31]. Traditional HPMC is soluble in water; thus, it is not suitable for a water and high humidity environment. It is usually added to liquid as a corrosion inhibitor. In the sustainable manufacturing applications, solid films were used to replace the solution-type inhibitor for the environmental-friendly considerations. Therefore, acetate, succinate, and phthalates were added to HPMC to obtain hydroxypropyl methylcellulose phthalate (HPMCP) and hydroxypropyl methylcellulose acetate succinate (HPMCAS). They not only preserve the characteristics of HPMC, but are also insoluble in water, and function well in strong acid environments. The material details of HPMCP and HPMCAS are listed in Table 1. The composition of high speed steel is listed in Table 2.

## 2. Results and Discussion

### 2.1. Film preparation and Characteristics Measurement

**Figure 1:**
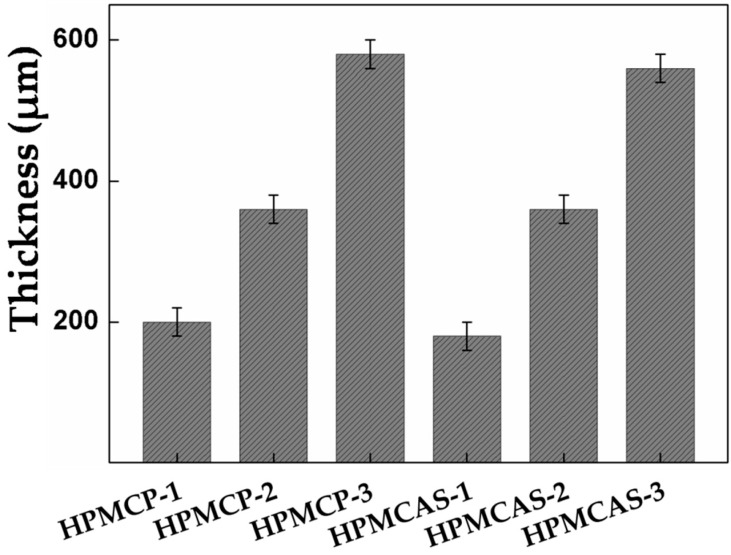
Thickness distributions of HPMCP and HPMCAS.

The film thickness can be controlled by precisely adjusting the drop amount, as shown in Figure 1. HPMCP-1, HPMCP-2 and HPMCP-3 correspond to 600, 1200 and 1800 μL, respectively. The corresponding thicknesses are 200, 360, and 580 μm, respectively. HPMCAS-1, HPMCAS-2 and HPMCAS-3 correspond to 600, 1200 and 1800 μL, respectively. The corresponding thicknesses are 180, 360, and 560 μm, respectively. The results show that the thickness of the HPMCP and HPMCAS films could be accurately adjusted and controlled.

**Figure 2:**
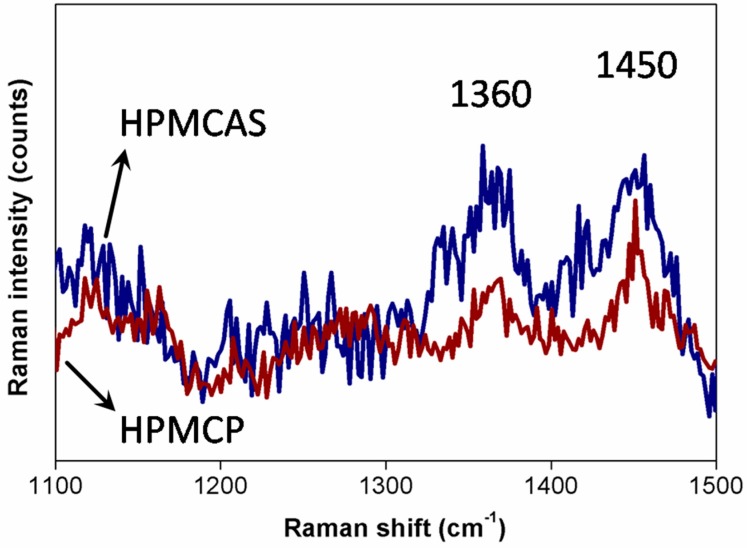
Raman spectroscopy graphs of HPMCP and HPMCAS.

Raman spectroscopy was utilized to investigate the material characteristics of HPMC derivatives. The Raman spectra of HPMCP and HPMCAS are presented in Figure 2. The characteristic peaks of HPMC are depicted, including those at 1360 cm^−1^ (COH bending) and 1450 cm^−1^ (CH_2_ twist) [32]. Moreover, a comparison of the two curves of HPMCP and HPMCAS shows that there is no obvious different absorption peak. It reflects that although phthalate, acetate, and succinate were added to HPMC to meet the requirements of acid and moisture resistance, HPMCP and HPMCAS preserved the structural properties of HPMC. Therefore, Raman spectroscopy could be used to assess the material, decomposability, and uniformity properties of HPMCP and HPMCAS [32,33].

### 2.2. Anti-Corrosion Behavior

**Figure 3:**
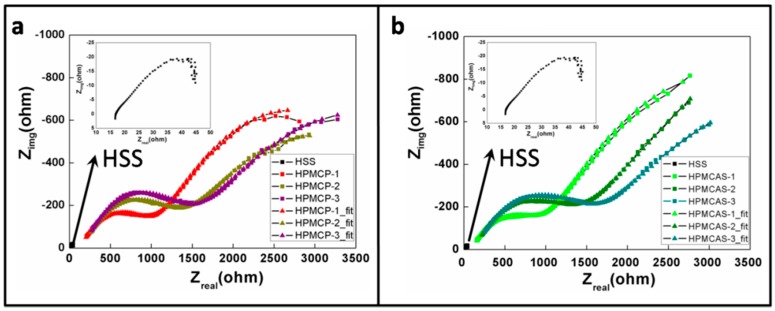
Nyquist plot of (**a**) HPMCP with varying thickness, where the inset is the Nyquist plot of uncoated high speed steel (HSS); (**b**) HPMCAS with varying thickness.

**Figure 4:**
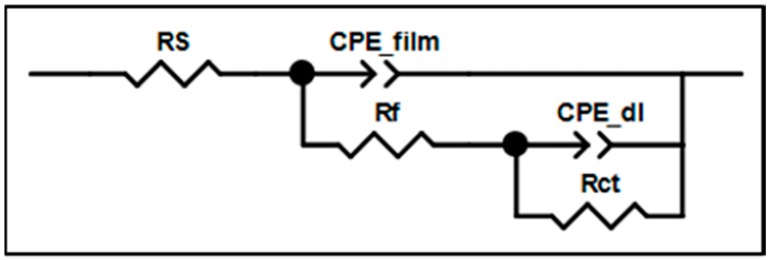
EIS equivalent circuit diagram.

Figure 3a shows the Nyquist plots of the four measured samples. The first sample is uncoated high speed steel (HSS). A series of samples are denoted HPMCP-1, HPMCP-2 and HPMCP-3, respectively. Figure 3b shows the Nyquist plots of HPMCAS with varying thickness. It can be seen that the left part was a semi-circle, and the right part was an incomplete semi-circle. Hence, it can be determined that the plot is comprised of two time constants. The equivalent circuit diagram shown in Figure 4 was used to simulate the actual conditions [34].

As shown in Figure 4, Rs represents the resistance of saline solution; Rf represents the resistance of the HPMC derivatives film; CPE_film is the capacitance of the HPMC film; Rct is the resistance between the steel and solution, also called the charge-transfer resistance; CPE_dl is the capacitance of the double electrode layer. CPE was used in this study as opposed to the capacitance in the traditional equivalent circuit model as there was an uneven current potential distribution; CPE would be more accurate according to previous studies [35,36,37].

In the equivalent circuit diagram, Rf corresponds to the left semi-circle in Figure 3; the larger the Rf, the larger the radius in the Nyquist plot, and the better the anti-corrosion ability. Rct corresponds to the right semi-circle. As the aim of the current study is to investigate the anti-corrosion ability of the film, we focus on Rf.

The original data was fitted with an equivalent circuit diagram, and the fitting data is shown in Table 2. The film resistance (Rf) of HPMCP-1, HPMCP-2, and HPMCP-3 are 989, 1260, and 1368 Ω, respectively. The film resistance of HPMCAS-1, HPMCAS-2, and HPMCAS-3 are 987, 1339 and 1535 Ω, respectively. For both materials, the film resistance increased with increasing film thickness, as did the penetration depth [38], indicating enhanced resistance ability with increasing film thickness.

By comparing the Rf for HPMCP and HPMCPAs, the results clearly demonstrate that HPMCP and HPMCAS were at the same scale. However, the increase in impedance for the HPMCAS can be attributed to it having a better hydrophilicity than HPMCP. The high capacitance, CPE_film, is related to the high extent at which water has penetrated the film [39]. Comparison of CPE_film-T for HPMCAS and HPMCP shows that the former had a larger CPE_film-T, i.e., greater moisture content. This is consistent with the experiment on contact angles. Compared to HPMCP, HPMCAS showed a smaller contact angle, namely, a better hydrophilic property. Higher hydrophilicity of HPMCAS compared to HPMCP and bare HSS may result from high-wettability. This leads to an increased concentration of corrosive substance on the HSS surface.

**Figure 5:**
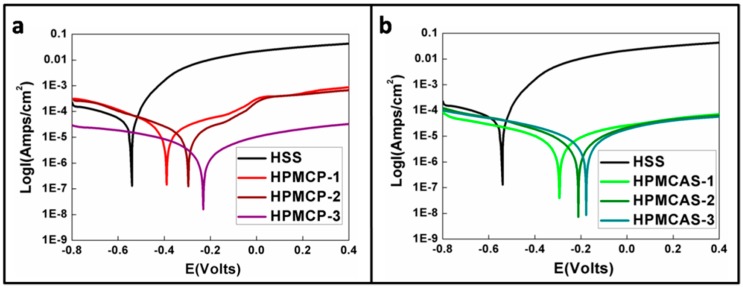
Polarization curve of (**a**) HPMCP with varying thickness; (**b**) HPMCAS with varying thickness.

Previous results show that HPMCP and HPMCAS films had demonstrated promising anti-corrosion behavior. The potentiodynamic polarization (PP) method was further used to record the variation in current and potential during the experiment. The polarization curves for HPMCP and HPMCAS with different thicknesses are shown in Figure 5. The curves are divided into cathodic and anodic polarization. Cathodicpolarization is the section before the lowest point, representing hydrogen reduction in the experiment: 2H^+^ + 2e^−^ → H_2_. Anodic polarization is the right section after the lowest point, representing metal oxidation in the experiment: M → M^n+^ + ne^−^.

The bottom point of the curve represents the corrosion potential. The corrosion current (Icorr) was measured using Tafel extrapolation. Within 50 mV of the corrosion potential, a linear region, called the Tafel region, was obtained. The tangent lines of cathodic polarization (slope βa) and anodic polarization (slope βc) intersect in the horizontal axis at the point of corrosion current (Icorr), which represents the corrosion rate. In the present study, Icorr was used to evaluate the anti-corrosion ability of the film [40,41], the data of which are shown in Table 3.

The electrochemical corrosion measurements of HSS, HPMCP and HPMCAS are shown in Table 4. The Tafel plots for the HPMCP yield corrosion potentials of Ecorr = −388.1, −294.9, and −230.5 mV for HPMCP-1, HPMCP-2, and HPMCP-3, respectively, which are more positive than that of the bare HSS, where Ecorr = −547.5 mV. Moreover, the corrosion current (Icorr) of HPMCP-1, HPMCP-2, and HPMCP-3 were 6.8, 5.2, and 0.8 μA/cm^2^, respectively, which are significantly lower than that of the HSS sample (26.3 μA/cm^2^).

The Tafel plots for the HPMCAS yield a corrosion potential of Ecorr = −294.4, −211.1, and −176.7 mV for HPMCAS-1, HPMCAS-2, and HPMCAS-3, respectively, which are more positive than that of the bare HSS. Moreover, the corrosion current (Icorr) for HPMCAS-1, HPMCAS-2, and HPMCAS-3 was 1.8, 1.4, and 1.7 μA/cm^2^, respectively, which are significantly lower than that of the HSS. From Table 3, it can be seen that for HPMCP and HPMCAS, the corrosion current decreased with increasing film thickness, indicating a reduced corrosion rate. Thus, there is a positive correlation between film thickness and the corrosion resistance performance.

Comparison of HPMCP-3 and HPMCAS-3 shows that the corresponding Icorr decreased considerably when we used the phthalate function group, suggesting the formation of hydrophobic properties. The electrochemical measurement results show that the HPMCP film provided better protection against corrosion of the HSS than the HPMCAS.

In terms of viscosity, the values for HPMCP and HPMCAS were 100 and 200 mPa·s, respectively. The high viscosity of HPMCAS resulted in poor film formation, causing defects and inferior smoothness of the film [42], and, therefore, poor corrosion resistance. HPMCP had low material viscosity, hydrophobic surface and low moisture content resulted in promising corrosion resistance performance.

## 3. Materials and Methods

### 3.1. Film Preparation and Characteristics Measurement

First, 10 g of HPMCP and HPMCAS powders (Shin-Etsu Chemical Co., Ltd., Tokyo, Japan) were mixed with 18 mL water and 72 mL alcohol. The solution was stirred at room temperature until all particles were dissolved. A micropipette was used to draw up 600–1800 μL of the mixed solution and to deposit it on polished high-speed steel. A film was then formed after 1-day rest under normal temperature and pressure conditions. A 3D scanner (Keyence, VK9710, Osaka, Japan) was used for the thickness measurement.

The Raman spectra were recorded using a micro-Raman spectrometer (Renishaw, New Mills, UK). The contact angles were measured using a First Ten Angstroms FTA-1000B (Portsmouth, VA, USA) at ambient temperature. Water droplets were carefully dropped onto the surface of the samples, and the contact angle was determined from the average of three measurements at various positions on the sample.

### 3.2. Anti-Corrosion Behavior

The corrosion potential and corrosion current of samples were electrochemically measured by the PP method. The working electrode was made of Teflon to grip the high speed steel test piece. A saturated calomel electrode was used as the reference electrode to measure the potential. The auxiliary electrode was a platinum electrode to conduct current. The output potential current was controlled and measured by a potentiostat. The electrolyte was a 0.5 M saline solution.

EIS were recorded on an AC Impedance Analyzer (HIOKI 3533-05, Nagano, Japan). The frequency range was 200,000–0.01 Hz, and the amplitude was 0.01 V, 10 points/decade.

## 4. Conclusions

(1) The corrosion resistance performance of green polymer material HPMC derivatives was demonstrated.
(2) Both EIS and PP suggested promising corrosion resistance performance of HPMCP and HPMCAS.
(3) The film thickness of HPMC derivatives was positively correlated the corrosion resistance ability.
(4) HPMCP has hydrophobic surface and low moisture content; thus, it provided better anti-corrosion protection than HPMCAS.

## Figures and Tables

**Table 1:** The compositions list of hydroxypropyl methylcellulose phthalate (HPMCP) and hydroxypropyl methylcellulose acetate succinate (HPMCAS).

Materials	Molecular Weight	Methoxy Content (%)	Hydroxypropoxy Content (%)	Phthalyl Content (%)	Acetyl Content (%)	Succinoyl Content (%)
HPMCP	37,900	20.0%~24.0%	6.0%~10.0%	21.0%~27.0%		
HPMCAS	18,000	20.0%~24.0%	5.0%~9.0%		5.0%~9.0%	14.0%~18.0%

**Table 2:** Composition of high speed steel.

Composition	C	Mn	Cr	W	V	Mo	Si	P	S	Ni	Cu	Fe
SKH51	0.82	0.24	4.20	6.50	2.05	5.78	0.23	0.02	0.01	0.08	0.12	79.95

**Table 3:** EIS fitting results and contact angles of HPMCP and HPMCAS coated electrodes.

Item/Index	Rs (Ω)	Rf (Ω)	CPE_film-T(F)	CPE_fim-P	Contact Angle (°/H_2_O)
HPMCP-1	128	989	3.49 × 10^−5^	0.40342	68.8
HPMCP-2	157	1260	1.85 × 10^−5^	0.42213	68.6
HPMCP-3	160	1368	1.34 × 10^−5^	0.44124	69.4
HPMCAS-1	90	987	5.49 × 10^−5^	0.38172	63.3
HPMCAS-2	115	1339	2.76 × 10^−5^	0.39806	65.0
HPMCAS-3	120	1535	2.42 × 10^−5^	0.39101	63.0

**Table 4:** Electrochemical corrosion measurement of HSS, HPMCP and HPMCAS coated electrodes.

Item/Index	−E_corr_ (mV)	I_corr_ (μA/cm^2^)	βa (mV/dec)	βc (mV/dec)
bare HSS	547.5	26.3	65.4	−159.9
HPMCP-1	388.1	6.8	124.9	−99.3
HPMCP-2	294.9	5.2	112.2	−119.9
HPMCP-3	230.5	0.8	117.5	−108.0
HPMCAS-1	294.4	1.8	119.5	−114.5
HPMCAS-2	211.1	1.4	105.5	−108.4
HPMCAS-3	176.7	1.7	102.6	−105.2

## Abbreviations

The following abbreviations are used in this manuscript:

NCKU	National Cheng Kung University
HPMC	hydroxypropyl methylcellulose
HPMCP	hydroxypropyl methylcellulose phthalate
HPMCAS	hydroxypropyl methylcellulose acetate succinate
HSS	high speed steel
PP	potentiodynamic polarization
